# A Systematic Analysis Identifies Key Regulators Involved in Cell Proliferation and Potential Drugs for the Treatment of Human Lung Adenocarcinoma

**DOI:** 10.3389/fonc.2021.737152

**Published:** 2021-09-28

**Authors:** Kai Wang, Man Zhang, Jiao Wang, Pan Sun, Jizhuang Luo, Haizhen Jin, Rong Li, Changqing Pan, Liming Lu

**Affiliations:** ^1^ Clinical Research Center, Shanghai Chest Hospital, Shanghai Jiao Tong University, Shanghai, China; ^2^ Department of Radiology, Xiangyang Hospital of Traditional Chinese Medicine, Hubei University of Traditional Chinese Medicine, Xiangyang, China; ^3^ Laboratory of Molecular Neural Biology, School of Life Sciences, Shanghai University, Shanghai, China; ^4^ Central Laboratory, Shanghai Chest Hospital, Shanghai Jiao Tong University, Shanghai, China; ^5^ Department of Thoracic Surgery, Shanghai Chest Hospital, Shanghai Jiao Tong University, Shanghai, China; ^6^ Department of Pulmonary Medicine, Shanghai Chest Hospital, Shanghai Jiao Tong University, Shanghai, China; ^7^ China Hospital Development Institute, Shanghai Jiao Tong University, Shanghai, China; ^8^ General Surgery Department, Shanghai Chest Hospital, Shanghai Jiao Tong University, Shanghai, China; ^9^ Shanghai Institute of Immunology, Shanghai Jiao Tong University School of Medicine, Shanghai, China

**Keywords:** lung adenocarcinoma, cell proliferation, protein–protein interaction, transcriptional regulation, prognosis

## Abstract

Lung adenocarcinoma (LUAD) is one of the most common and malignant cancer types. Abnormal cell proliferation, exemplified by cell cycle and cell division dysregulation, is one of the most prominent hallmarks of cancer and is responsible for recurrence, metastasis, and resistance to cancer therapy. However, LUAD-specific gene regulation and clinical significance remain obscure. Here, by using both tissues and cells from LUAD and normal lung samples, 434 increased and 828 decreased genes of biological significance were detected, including 127 cell cycle-associated genes (95 increased and 32 decreased), 66 cell division-associated genes (56 increased and 10 decreased), and 81 cell proliferation-associated genes (34 increased and 47 decreased). Among them, 12 increased genes (*TPX2*, *CENPF*, *BUB1*, *PLK1*, *KIF2C*, *AURKB*, *CDKN3*, *BUB1B*, *HMGA2*, *CDK1*, *ASPM*, and *CKS1B*) and 2 decreased genes (*TACC1* and *MYH10*) were associated with all the three above processes. Importantly, 2 (*CDKN3* and *CKS1B*) out of the 11 increased genes (except *HMGA2*) are previously uncharacterized ones in LUAD and can potentially be prognostic markers. Moreover, *PLK1* could be a promising therapeutic target for LUAD. Besides, protein–protein interaction network analysis showed that *CDK1* and *CDC20* were the hub genes, which might play crucial roles in cell proliferation of LUAD. Furthermore, transcriptional regulatory network analysis suggested that the transcription factor E2F1 could be a key regulator in controlling cell proliferation of LUAD *via* expression modulation of most cell cycle-, cell division-, and cell proliferation-related DEGs. Finally, trichostatin A, hycanthone, vorinostat, and mebeverine were identified as four potential therapeutic agents for LUAD. This work revealed key regulators contributing to cell proliferation in human LUAD and identified four potential therapeutic agents for treatment strategy.

## Introduction

Lung adenocarcinoma (LUAD) is one of the most common and malignant cancer types, accounting for more than 50% of lung cancer. Targeted therapy and immunotherapy are two of the major ways used in LUAD treatment. The therapeutic targets usually include growth factor receptors of lung cancer cell membrane (e.g., EGFR), specific kinases (e.g., ALK and ROS1) in signal transduction pathways, and specific genes related to proliferation, division, invasion, and metastasis of LUAD (1). By contrast, in immunotherapy, autologous immune cells were modified *via* targeting some key regulator genes involved in cancer pathogenesis and development. After activation and proliferation *in vitro*, they were reinfused to patients to stimulate anti-tumor immune effect or kill tumor directly. For example, blocking the immune checkpoint-related genes PD-1, PD-L1, and PD-L2 were adopted in anti-PD therapy to enhance T cell-mediated anti-tumor immunity (2–4), while the CAR-T cells kill tumors by specifically targeting their extracellular antigens, such as CD19 and EGFR (5). Despite these marvelous achievements, cytotoxic effects and therapeutic effects still need to be improved. A precise understanding of the molecular characteristics should help us discover the key regulators of cancer pathogenesis, biomarkers for cancer diagnosis and prognosis, and targets for cancer therapy.

Cell proliferation was the major cause of tumor recurrence and metastasis, and resistance to cancer therapy (6, 7). Cell cycle- and cell division-associated genes are most relevant to cell proliferation of various cancers (8). Many studies have been carried out to detect the related molecular signatures and prognostic markers in lung cancer. Based on microarray, some common markers of proliferation (such as E2F1 and MCM2-MCM6) were detected by comparing normal and tumor samples, and these genes were commonly associated with the expression of a core set of cell cycle-associated genes (8). Silencing of E2F1 by long non-coding RNA could suppress tumor growth in LUAD patients, but the downstream target genes of E2F1 were not clear (9). In the A549 human lung cancer cell line, AMMECR1 was found to play a role in inhibiting apoptosis and promotes cell-cycle progression and proliferation (10). Variant CD44 expression was enriched in LUAD cell lines with CSC characteristics and more proliferative (11). MCM4 was reported as a marker for proliferation for non-small cell lung cancer (12). However, more driver factors involved in these processes are still to be discovered, and how they worked together to promote cell proliferation in human LUAD is still not clear from a systemic view.

In this study, we identified differentially expressed genes (DEGs) from LUAD and normal lung samples, predicted cell cycle-, cell division-, and cell proliferation-associated prognostic markers, determined key regulators involved in cell proliferation, and investigated their protein–protein interaction (PPI) and transcriptional regulatory (TR) networks from a system view. This study provided new clues for our understanding of human LUAD pathogenesis, as well as biomarkers for diagnosis and prognosis, and potential targets for immunotherapy.

## Materials and Methods

### Raw RNA-Seq Data Collection and Pre-Processing

The NCBI Gene Expression Omnibus (GEO, https://www.ncbi.nlm.nih.gov/geo/) (13) database hosts various gene expression data that could be used for re-analyzing. Raw RNA-Seq data of LUAD tissues (GSE40419) and cells (GSE66727, GSE86337, and GSE57148) with the accession number were downloaded from NCBI GEO website that links to Sequence Read Archive (SRA). SRA Toolkit (version 2.9.2) was used to convert all SRA data to FASTQ data. Low-quality bases (Q < 20) and adaptors at both 5’ and 3’ ends were trimmed by Trimmomatic (version 0.38) (14). Paired-ended reads with at least 35 bp were reserved. HISAT2 (version 2.1.0) (15) was used to align all cleaned reads to the human transcriptome assembly GRCh38.p12. BEDTools (Version 2.27.1) (16) and a localized Perl script were used to calculate reads number and Reads Per Kilobase per Million mapped reads (RPKM) values of each gene, respectively.

### Principal Component Analysis

Gene Cluster 3.0 (http://bonsai.hgc.jp/~mdehoon/software/cluster/index.html) (17) is an open-source clustering software that was originally developed by Michael Eisen of Berkeley Lab. It encapsulated various useful methods that can be used to analyze gene expression data, such as hierarchical clustering, k-means clustering, PCA, and 2D self-organizing maps. Here, PCA was performed by Gene Cluster 3.0 based on all genes. The first two principal components were plotted.

### DEGs and Functional Enrichment

All DEGs were detected using an absolute fold change (FC) threshold of ≥2.0 and a *p*-value of ≤0.05. The Database for Annotation, Visualization and Integrated Discovery (DAVID) tool (https://david.ncifcrf.gov/) (18) is a toolkit for exploring significant biological meaning from a given list of genes. In this study, the functions of all DEGs were annotated using the DAVID v6.8. The three major enriched GO terms in molecular function (MF), biological process (BP), and cellular component (CC) categories as well as KEGG pathways of all DEGs were applied.

Besides, DEGs associated with cell cycle, cell division, and cell proliferation were extracted from the AmiGO (http://amigo.geneontology.org/amigo) (19) database according to their GO annotation. The AmiGO is an online tool that provides comprehensive ontology annotation information and powerful searching tools for protein functional mining. Specifically, the proteins annotated with the GO terms cell cycle (GO: 0007049), cell division (GO: 0051301), and cell proliferation (GO:0008283) as well as their child GO terms were retrieved.

### DEGs Validation by qRT-PCR Experiment

Total RNA was extracted from both cells (95D and H1299) and patient tissues (P158, P162, P167, and P168) using TRIzol Reagent (Thermo Fisher Scientific) and reverse transcribed into cDNA using oligo-dT shortly. PCR primers (**Table 1**) were designed using PrimerBank (20) and synthesized by Sangon Biotech (Shanghai, China). PCR reactions were carried out on an ABI ViiA7 thermocycler (Applied Biosystems) for 40 cycles (95°C for 10s, 55°C for 15s, and 65°C for 15s). Relative fold changes were calculated based on Ct values, and GAPDH was used as internal control.

**Table 1:** Primers used for qRT-PCR assay.

Gene Symbol	Forward Primer	Reverse Primer
*TPX2*	ATGGAACTGGAGGGCTTTTTC	TGTTGTCAACTGGTTTCAAAGGT
*CENPF*	CTCTCCCGTCAACAGCGTTC	GTTGTGCATATTCTTGGCTTGC
*BUB1*	TGGGAAAGATACATACAGTGGGT	AGGGGATGACAGGGTTCCAAT
*PLK1*	AAAGAGATCCCGGAGGTCCTA	GGCTGCGGTGAATGGATATTTC
*KIF2C*	CTGTTTCCCGGTCTCGCTATC	AGAAGCTGTAAGAGTTCTGGGT
*AURKB*	CAGTGGGACACCCGACATC	GTACACGTTTCCAAACTTGCC
*CDKN3*	TCCGGGGCAATACAGACCAT	GCAGCTAATTTGTCCCGAAACTC
*BUB1B*	AAATGACCCTCTGGATGTTTGG	GCATAAACGCCCTAATTTAAGCC
*HMGA2*	ACCCAGGGGAAGACCCAAA	CCTCTTGGCCGTTTTTCTCCA
*CDK1*	AAACTACAGGTCAAGTGGTAGCC	TCCTGCATAAGCACATCCTGA
*ASPM*	GGCCCTAGACAACCCTAACGA	AGCTTGGTGTTTCAGAACATCA
*CKS1B*	TATTCGGACAAATACGACGACG	CGCCAAGATTCCTCCATTCAGA
*TACC1*	AGGGGCAGTGATCTCCCAG	TTTCTGACCACATGACGTGGA
*MYH10*	TGGTTTTGAGGCAGCTAGTATCA	AGTCCTGAATAGTAGCGATCCTT
*E2F1*	ACGCTATGAGACCTCACTGAA	TCCTGGGTCAACCCCTCAAG
*GAPDH*	GGAGCGAGATCCCTCCAAAAT	GGCTGTTGTCATACTTCTCATGG

Note that GAPDH was used as a negative control.

### Protein–Protein Interaction Network

The BioGRID database (https://thebiogrid.org/) (21) deposited biomedical interaction with data comprehensive curation efforts. Besides, the IntAct Molecular Interaction Database (https://www.ebi.ac.uk/intact/) (22) is a freely available and open-source database for molecular interaction data. In this study, PPIs of human and mouse were collected from both BioGRID and IntAct. Interaction data were merged according to gene name to gene name pairs. The first neighbors of cell cycle-, cell division-, and cell proliferation-related DEGs and the PPIs between them were extracted from the merged interaction dataset. Only reliable interactions were reserved, including those detected by at less two different experimental methods, or curated from at less two literatures, or existed in both human and mouse. The network was displayed by Cytoscape 3.8.0 (23).

### Transcriptional Regulatory Network

Embryonic Stem Cell Atlas from Pluripotency Evidence (ESCAPE, http://www.maayanlab.net/ESCAPE/) (24) is a TR database that is originally designed for pluripotency and self-renewal studies, but most interactions should be conserved and could also be used in cancer studies. All interactions in this database were obtained from human and mouse embryonic stem cells using high-throughput screening methods. Transcriptional Regulatory Relationships Unravelled by Sentence-based Text-mining (TRRUST, https://www.grnpedia.org/trrust/) (25) is a manually curated database containing human and mouse TF–target data. All interactions were detected by small-scale experiments. The Encyclopedia of DNA Elements (ENCODE, https://www.encodeproject.org/) (26) is a comprehensive database of functional elements for humans. It includes both genes and their regulation information. TF–target interactions in ESCAPE, TRRUSTv2, and (score ≥300) were retrieved. TFs that differentially expressed between cancer and normal samples were selected. Only these interactions that presented in at least two of the three databases were selected. The first TF neighbors of the cell cycle-, cell division-, and cell proliferation-related DEGs and the TR interactions between them were used for network construction. The network was displayed by Cytoscape 3.8.0.

### Connectivity Map Analysis

The Connectivity Map (CMAP, www.broad.mit.edu/cmap) is a drug discovery database (27, 28). Based on the gene expression change information, it infers functional associations between drugs, genes, and diseases by employing pattern-matching algorithms. The correlation between gene expression signatures (a list of DEGs of biological interest) and bioactive molecules of cMAP was estimated by a connectivity score. A positive score denotes a simulative relationship and a negative score reflects a repressed relationship. The gene symbols were converted to corresponding Affymetrix probe sets using the “Batch Query” tool on NetAffx (http://www.affymetrix.com/analysis/netaffx/index.affx) and selecting “HG-U133A” from the “Select a GeneChip Array” menu. The probe setlists of upregulated genes and downregulated genes were loaded into the cMAP web server as query signatures.

## Results

### The Transcriptome Profiling, DEGs, and Functional Annotation

**Figure 1:**
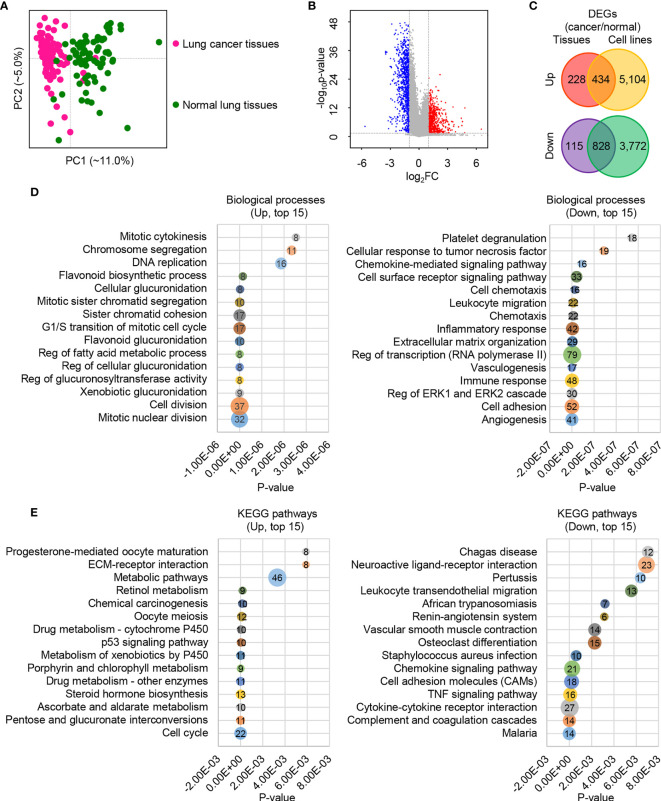
The global expression profiles of human lung cancer cells/tissues and adjacent lung normal cells/tissues. **(A)** The first two principal components plot of LUAD tissues *via* PCA. **(B)** Volcano plot of genes between human lung cancer tissues and adjacent lung normal tissues. The absolute FC of ≥2.0 and *p*-value of ≤0.05 were used. **(C)** Venn diagram plot of DEGs based on tissues and cells with the absolute FC of ≥ 2.0 and *p*-value of ≤ 0.05. **(D)** Bubble diagram plot of the top 15 enriched biological processes for the upregulated (left) and downregulated (right) DEGs. **(E)** Bubble diagram plot of the top 15 enriched KEGG pathways for the upregulated (left) and downregulated (right) DEGs.

To determine the global gene expression differences between LUAD and normal samples, we collected raw RNA-Seq data from the NCBI GEO database. These samples were collected from both tissues (GSE40419) (29) and cells (GSE66727, GSE86337 and GSE57148) (30–32). Combining the two different types of data ensures the robustness of DEGs detection between LUAD and normal lung samples. We first surveyed the global gene expressions in patient tissues. Principal component analysis (PCA) showed that PC1 separated the cancer tissues (pink nodes in the left panel) from normal tissues (green nodes in the right panel), as can be seen from **Figure 1A**. When a fold change (FC) of ≥2.0 and a *p*-value of ≤0.05 were used, we detected a total of 662 upregulated genes (red points in the right panel) and 943 downregulated genes (blue points in the left panel) in cancer tissues (**Figures 1B, C** and **Supplementary Table S1**). In contrast, the cell lines showed much larger variation when compared with tissues; 5,538 upregulated genes and 4,600 downregulated genes were detected in cancer cells (**Figure 1C** and **Supplementary Table S1**). This difference reflects that cancer tissues have much more heterogeneity than cell lines. Among the commonly regulated genes, 434 were upregulated and 828 were downregulated (**Figure 1C**). Our subsequent analysis was based on these commonly regulated genes, which are more reliable than only using a single type of DEG dataset.

The Database for Annotation, Visualization and Integrated Discovery (DAVID) v6.8 (https://david.ncifcrf.gov/) (18) was employed to determine the function of these commonly regulated genes. Many upregulated DEGs were enriched in the cell cycle-associated processes, such as mitotic nuclear division, cell division, G1/S transition of mitotic cell cycle, and mitotic cytokinesis (**Figure 1D**, left panel and **Supplementary Table S2**). Also, some material and energy metabolism-associated biological processes were enriched, such as xenobiotic glucuronidation, negative regulation of glucuronosyl transferase activity, negative regulation of cellular glucuronidation, negative regulation of fatty acid metabolic process, and flavonoid glucuronidation (**Figure 1D**, left panel and **Supplementary Table S2**). Similarly, cell cycle, pentose and glucuronate interconversions, ascorbate and aldarate metabolism, and metabolic pathways were among the top 15 enriched pathways (**Figure 1E**, left panel and **Supplementary Table S2**). These findings are consistent with our knowledge about cancer cells that their cell proliferation is much more rapid than in normal cells (33–35), and these processes are material and energy dependent. On the contrary, genes in cell adhesion, extracellular matrix organization, immune response, inflammatory response, chemotaxis, and chemokine-mediated signaling pathway-related biological processes were suppressed (**Figure 1D**, right panel and **Supplementary Table S2**). Likewise, the top 15 prioritized pathways surveyed showed that the downregulated genes were enriched in cell adhesion molecules (CAMs), complement and coagulation cascades, cytokine–cytokine receptor interaction, and chemokine signaling pathway (**Figure 1E**, right panel and **Supplementary Table S2**). The depression of cell adhesion-related genes reflects a higher cell fluidity of LUAD than normal cells, which may be involved in cancer progression, metastasis, and affect anti-cancer therapeutic potential (36–38). The low expression of immune and inflammatory response-related genes reflects a vulnerable immune system of LUAD (39–41).

### DEGs Associated With Cell Cycle, Cell Division, and Cell Proliferation

**Figure 2:**
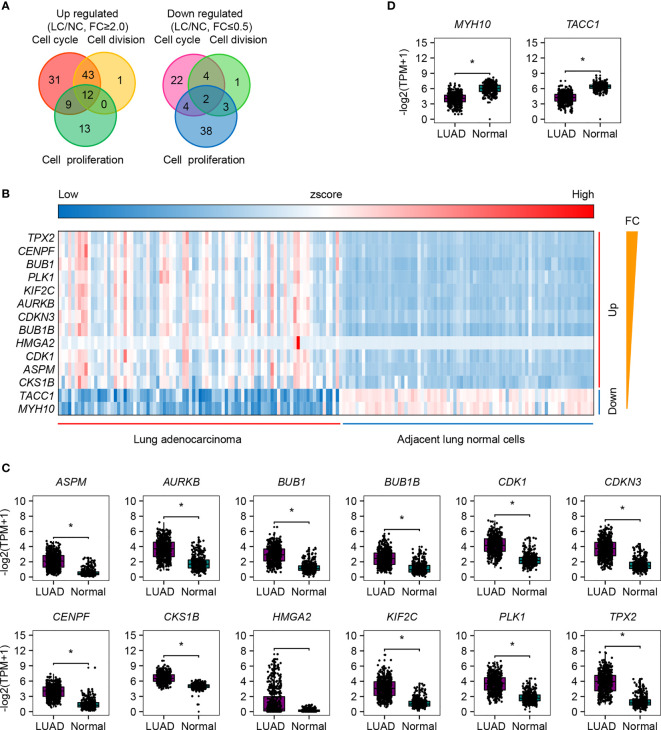
The up- and downregulated genes that are involved in cell cycle, cell division, and cell proliferation. **(A)** Venn diagram plot of the up- and downregulated genes that are involved in cell cycle, cell division, and cell proliferation. **(B)** Heatmap of the 12 upregulated and 2 downregulated genes involved in all the above three processes. **(C)** Expression validation of the 12 upregulated DEGs by GEPIA2. Reads box is the expression of LUAD samples; gray box is the expression of normal samples. **(D)** Expression validation of the two downregulated DEGs by GEPIA2. Reads box is the expression of LUAD samples; gray box is the expression of normal samples. *p.adj ≤ 0.05.

**Figure 3:**
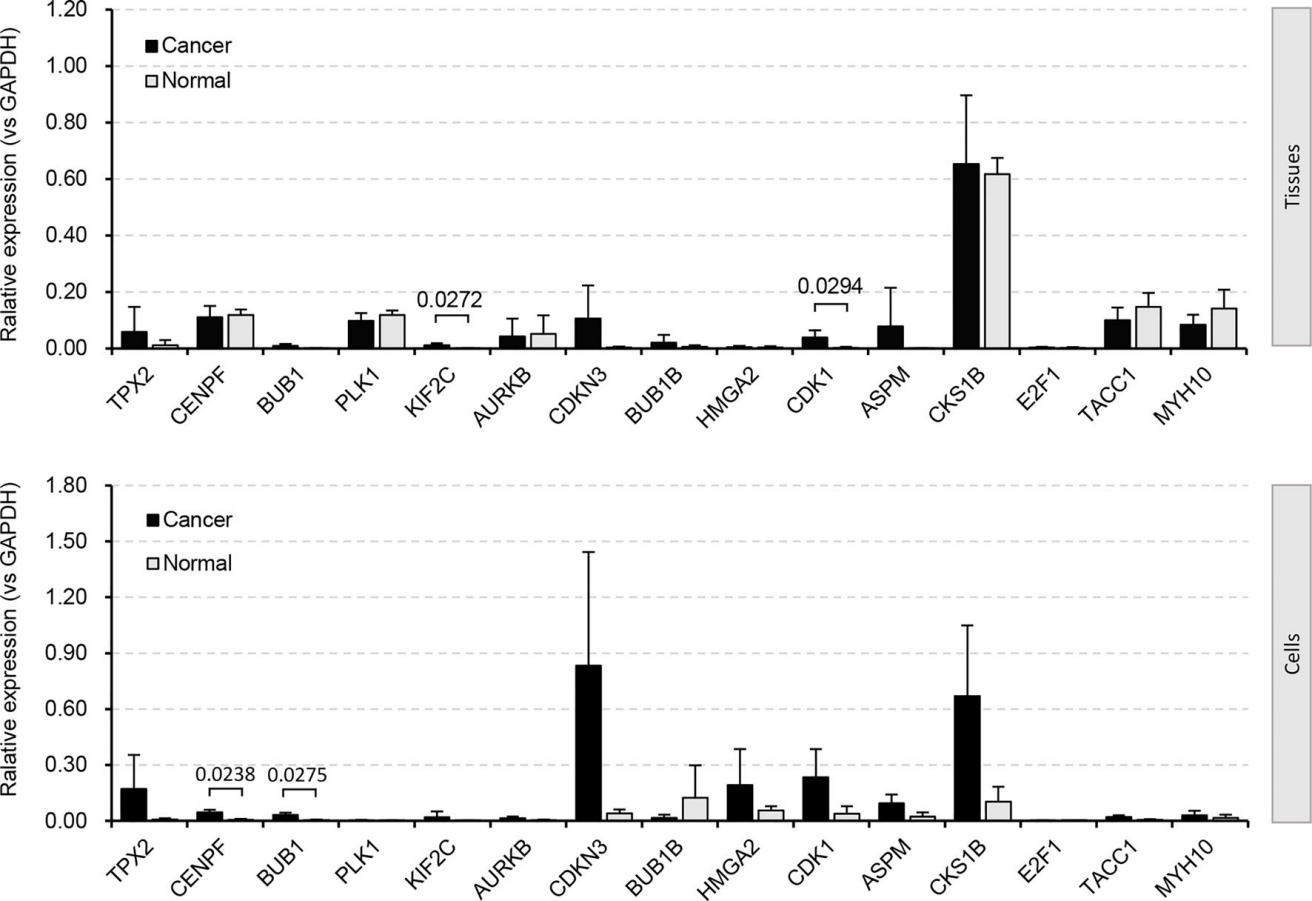
DEGs validated by qRT-PCR experiment. Four biological repetitions were applied to patient tissues; two biological repetitions and two technical repetitions were applied to cell lines. Human tumor-adjacent tissues and lung fibroblast cells (MRC-5) were used for control, respectively.

We paid special attention to the DEGs that are involved in cell cycle, cell division, and cell proliferation (**Figure 2A** and **Supplementary Figures S1**-**S3**), because they are strongly associated with tumor recurrence and metastasis, and usually considered as candidate targets for immunotherapy. Ninety-five increased and 32 decreased DEGs were associated with cell cycle, 56 increased and 10 decreased DEGs were associated with cell division, and 34 increased and 47 decreased DEGs were associated with cell proliferation (**Figure 2A**). Among them, 12 upregulated (*TPX2*, *CENPF*, *BUB1*, *PLK1*, *KIF2C*, *AURKB*, *CDKN3*, *BUB1B*, *HMGA2*, *CDK1*, *ASPM*, and *CKS1B*) and 2 downregulated (*TACC1* and *MYH10*) genes participated in all three processes (**Figures 2A, B**). The significant differential expression of all 12 upregulated (**Figure 2C**) genes except *HMGA2* and 2 downregulated (**Figure 2D**) genes was confirmed by the Gene Expression Profiling Interactive Analysis (GEPIA2, http://gepia2.cancer-pku.cn) (42). The additional qRT-PCR experiment was performed (**Figure 3**). A significant upregulation of KIF2C and CDK1 was observed in four patients’ tissues (P158, P162, P167, and P168), and a significant upregulation of *CENPF* and *BUB1* was observed in two cell lines (H1299 and 95D). Some other genes were also upregulated in patients’ tissues or cell lines, for example, *TPX2*, *CDKN3*, *HMGA2*, *ASPM*, and *CKS1B*. However, due to the heterogeneity of limited samples, no significance was detected. The qRT-PCR method estimates gene expression by detecting fluorescence intensity, while the RNA-Seq technology directly reflects the reads count in cells. Besides, the large sample size of the RNA-Seq dataset should eliminate internal errors raised by diversity to a more acceptable level. We thus performed further analyses based on the RNA-Seq result.

### Novel Prognostic Markers for LUAD

**Figure 4:**
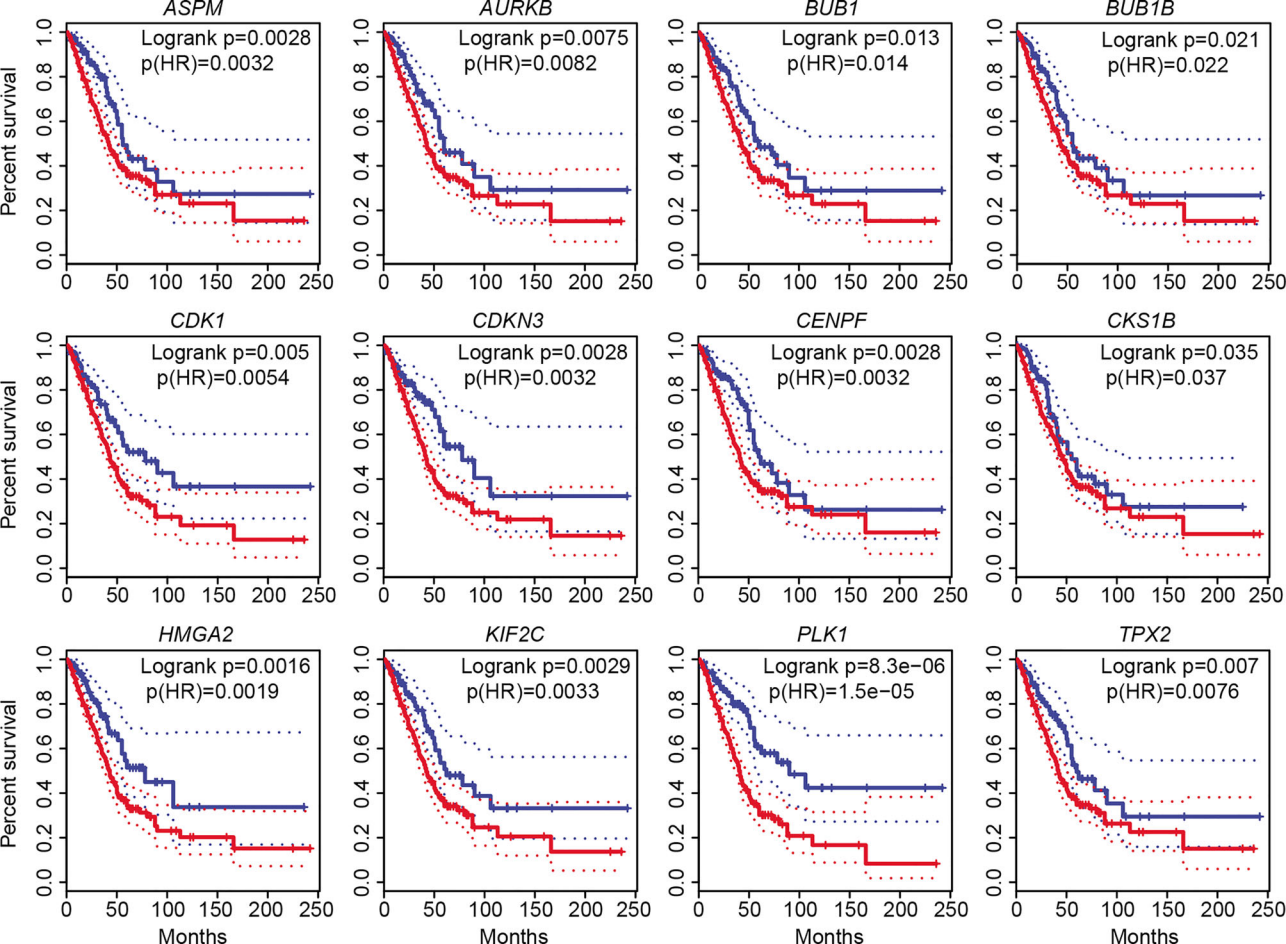
Overall survival (OS) analysis of 12 increased DEGs involved in cell cycle, cell division, and cell proliferation. The high-expression group was represented by red colors, and the low-expression group was represented by blue colors. Both high and low cutoffs were set to 25%, *n* (high) = 359, *n* (low) = 120. HR, hazard ratio.

To determine whether the above upregulated DEGs involved in cell cycle, cell division, and cell proliferation could be used as prognostic markers for LUAD diagnosis, we performed overall survival (OS) analysis by employing GEPIA2. All 11 genes (*TPX2*, *CENPF*, *BUB1*, *PLK1*, *KIF2C*, *AURKB*, *CDKN3*, *BUB1B*, *CDK1*, *ASPM*, and *CKS1B*), as well as *HMGA2*, showed significant difference (*p* ≤ 0.05) between high and low groups (**Figure 4**). Obvious differences were found before 60 months for 10 genes (*TPX2*, *CENPF*, *BUB1*, *PLK1*, *AURKB*, *CDKN3*, *BUB1B*, *CDK1*, *ASPM*, *and CKS1B*). No significance was found for the two downregulated genes (*TACC1* and *MYH10*). The increased expression of these cell cycle-, cell division-, and cell proliferation-associated genes in LUAD tissues with a poor prognosis forecasts crucial roles in malignant proliferation and carcinogenesis. All the 11 significantly increased genes could be potential biomarkers for the diagnosis of cell proliferation. Among them, *CDKN3* and *CKS1B* are previously uncharacterized ones in LUAD, which could be promising prognostic markers. Both expression and survival situation of the serine/threonine-protein kinase PLK1 were different between patients with LUAD and normal humans, suggesting that *PLK1* could be a therapeutic target for LUAD treatment.

### Cell Proliferation-Associated Protein-Protein Interaction Network in LUAD

**Figure 5:**
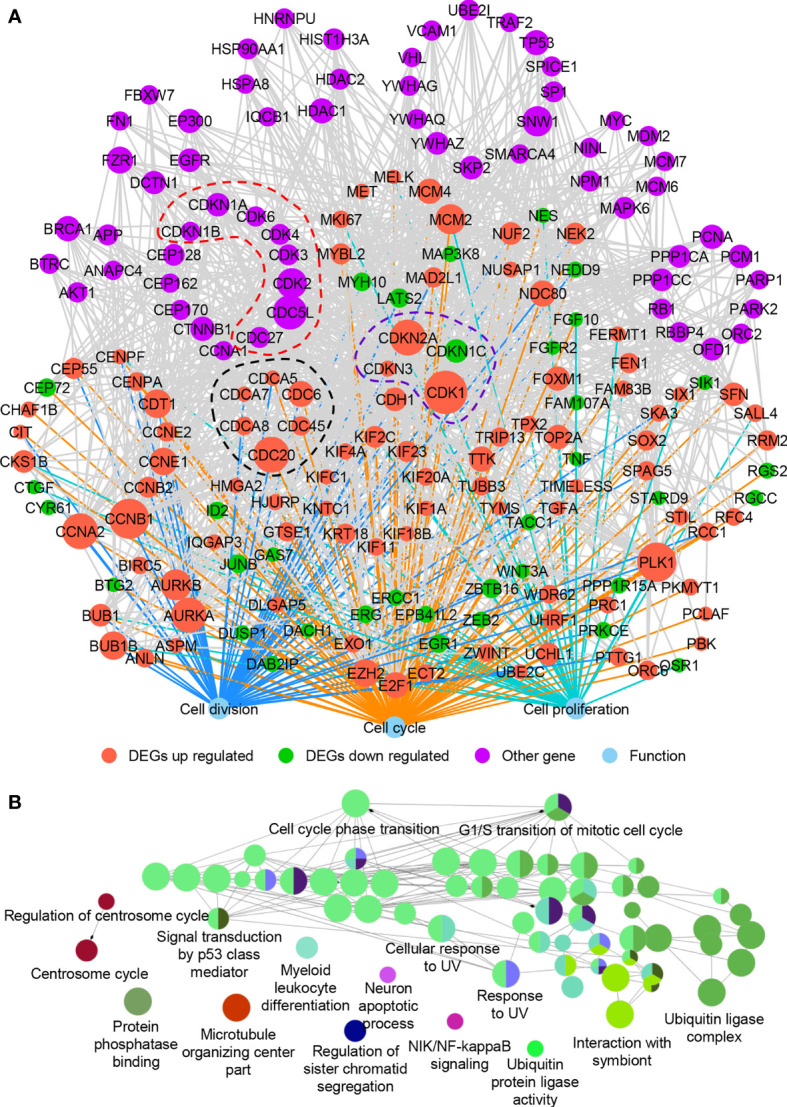
The protein–protein interaction network of DEGs involved in cell cycle, cell division, and cell proliferation. **(A)** The protein–protein interaction network. **(B)** ClueGO functional annotation of 61 other genes (purple nodes in **A**) that interacted with the up- and downregulated DEGs in **(A)**. A *p*-value of ≤0.01 was used, and experimental validated biological processes (BP), cellular components (CC), and molecular functions (MF) were plotted.

It is widely recognized that hub proteins in a protein interaction network interacted with many other partners; in other words, they process high connectivity than other proteins (43). To understand how the cell cycle-, cell division-, and cell proliferation-related DEGs (all genes in **Figure 2A**) worked together to promote the proliferation of LUAD cells, we examined their PPIs from the public database BioGRID (21) and IntAct (22). The network was constructed using the first neighbors, as described in the methods. These proteins were found to play more crucial roles than other proteins. Network hub analysis showed that the connection degree (as indicated by the node size) of the nodes *CDK1*, *CCNB1*, *PLK1*, *CCNA2*, *CDC20*, *CDKN2A*, *AURKA*, *CDC5L*, *AURKB*, *SNW1*, *MCM2*, and *CDK2* are ≥20, suggesting crucial roles of these genes in the network (**Figure 5A**).

The network module is a cohesively linked group of genes or their products (represented by nodes in the graph), which densely interacted internally and sparsely linked with the rest of the network (44). Network module analysis is a powerful approach to identify a group of genes that are known to often work together in common biological processes or shared molecular pathways. Previous studies showed that genes of the same families usually formed network modules (45). Inspired by this, we examined genes of the same families in this network and found that cell division cycle (CDC)- and cyclin-dependent kinase (CDK)-associated genes enriched, as indicated by the nodes in dotted black line (DEGs of CDC-associated genes, including *CDC6*, *CDC20*, *CDC45*, *CDCA5*, *CDCA7*, and *CDCA8*), dotted purple line (DEGs of CDK-associated genes, including *CDK1*, *CDKN1C*, *CDKN2A*, and *CDKN3*), and dotted red line (non-DEGs of CDC- and CDK-associated genes, including *CDC27*, *CDC5L*, *CDK2*, *CDK3*, *CDK4*, *CDK6*, *CDKN1A*, and *CDKN1B*) (**Figure 5A**). Almost all the CDC- and CDK-associated DEGs were increased in LUAD except *CDKN1C*. The hub genes *CDK1* and *CDC20* were also observed in the network modules (**Figure 5A**). Based on mouse and human models, CDK1 was found strongly associated with the progression of lung cancer and suggested as a potential prognostic biomarker (46, 47). CDC20 together with CENPA, CDK1, AURKA, and TPX2 were also diagnostic biomarkers in LUAD, and elevated mRNA levels of CDC20 were correlated with poor prognosis of lung cancer (48, 49). All these suggested that the CDC- and CDK-associated proteins play crucial roles in cell proliferation of LUAD. Besides, some genes associated with chromosome programming (such as *EP300*, *HDAC1*, *HDAC2*, *TP53*, *and MYC*) and post-translational modification (such as *HSP90AA1* and*HSPA8*) were found in the network (**Figure 5A**). The cell cycle- and cell division-associated genes may cooperate with these transcription regulators to promote the cell proliferation and progress of LUAD. However, the expression of these transcription regulators did not change significantly, indicating that they may play roles in translation or post-translation level.

To further verify the functions of proteins in the network, ClueGO functional analysis was carried out (**Figure 5B**). Many proteins related to the cell cycle in the network were also related to signal transmission by p53 class mediator, cellular response to UV, and ubiquitin ligase complex, further proving their roles in cell proliferation of LUAD. UV signals could lead to abnormal miRNA expression in human liver cancer cells (50) and p53 status (51). Ubiquitin ligase also played roles in cancers (52), which was associated with cell cycle and mitosis (53). All these revealed the close relationship between cell cycle-associated genes and cancer development.

### E2F1 Mediates the Expression of Most DEGs in LUAD

**Figure 6:**
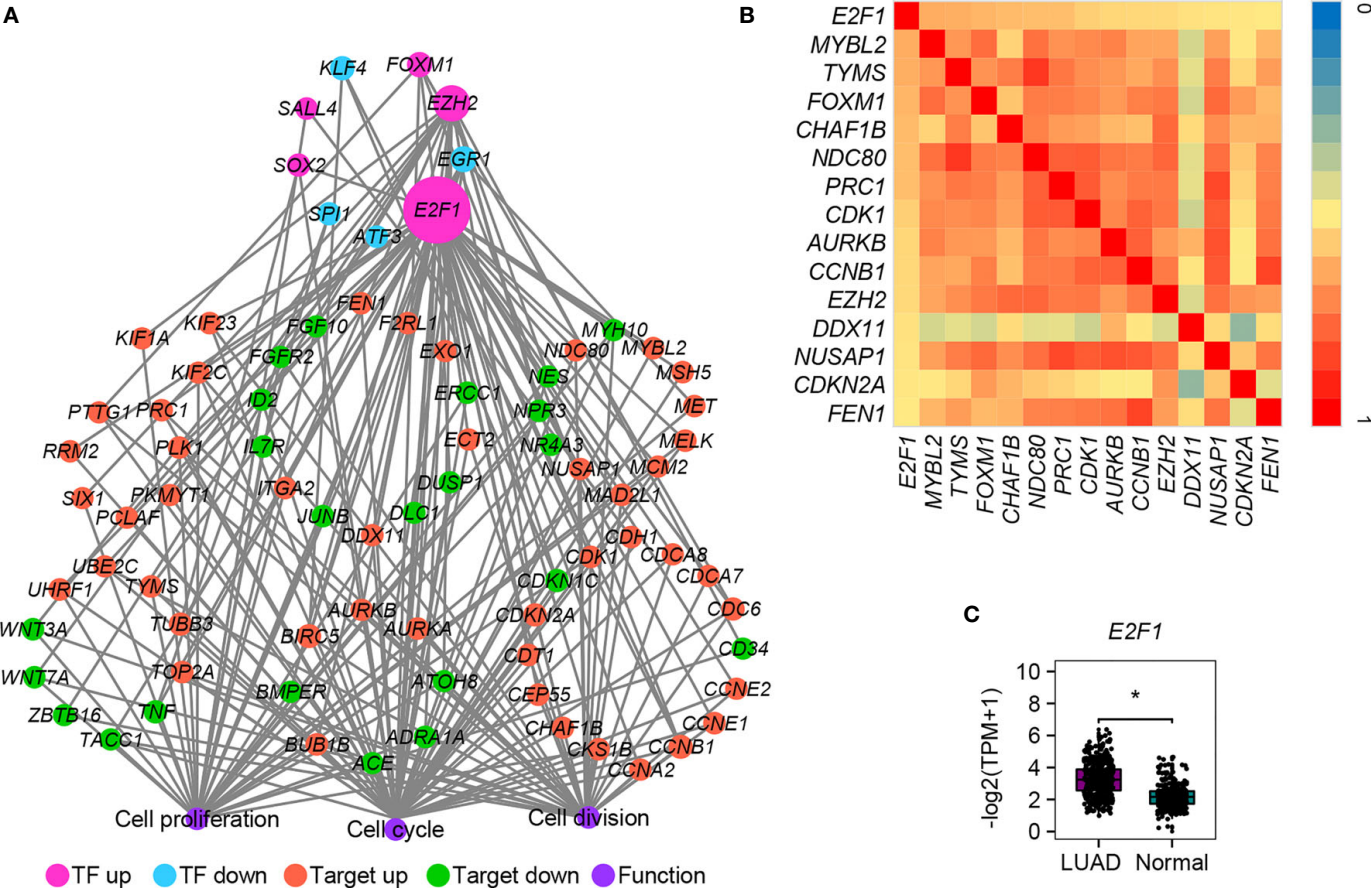
The transcriptional regulatory network of DEGs involved in cell cycle, cell division, and cell proliferation. **(A)** The TF–target interaction network. **(B)** Heatmap of Pearson correlation coefficient between E2F1 and its targets. The correlation coefficient was calculated by the *cor* function in R packages. Only the pairs with absolute values ≥ 0.5 were shown and no negative correlation was detected. **(C)** Expression verification of E2F1 between human lung cancer cells and adjacent lung normal tissues by GEPIA2. *p.adj ≤ 0.05.

**Figure 7:**
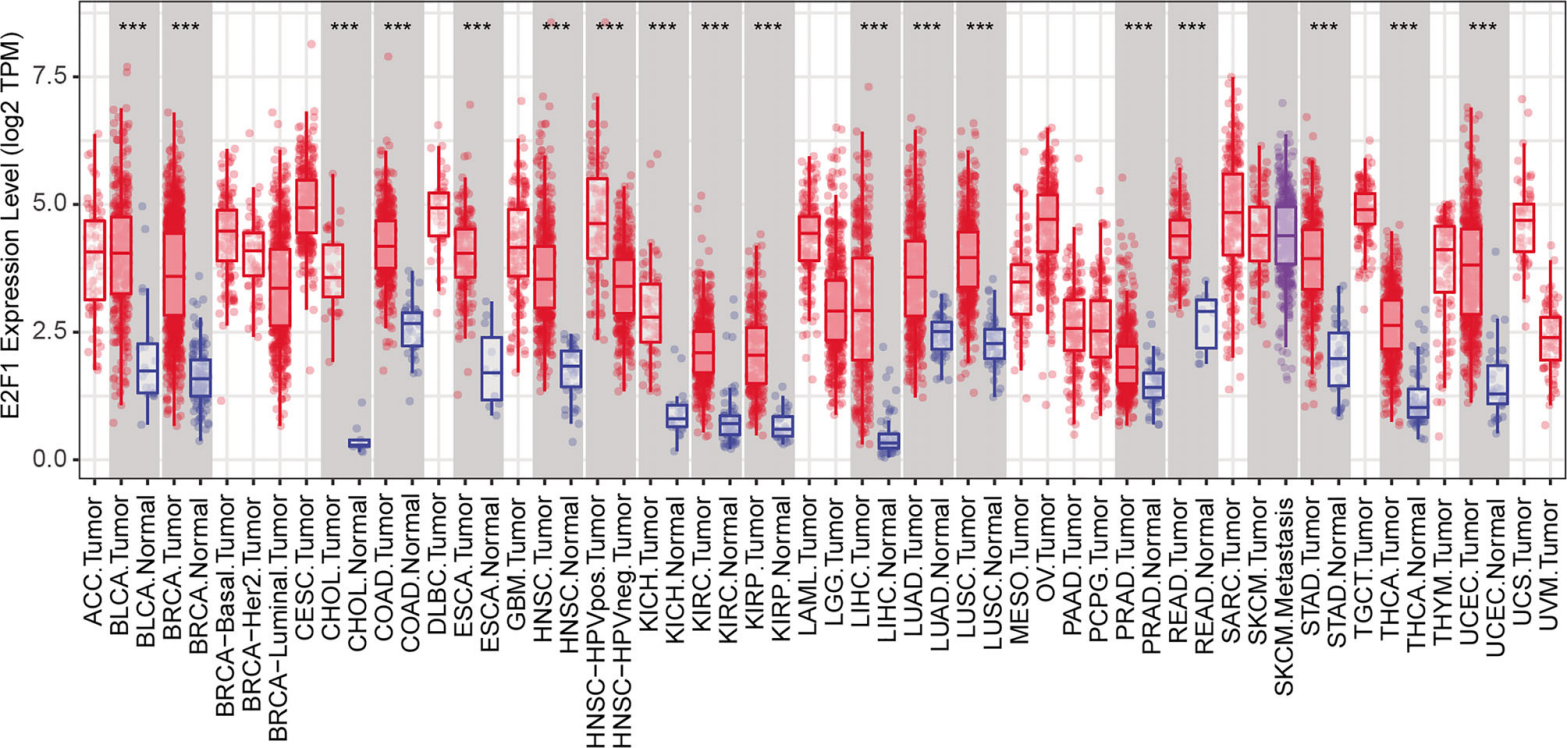
Conserved expression pattern of E2F1 among various cancers. ***p.adj ≤ 0.001.

We also determined to find out whether there are some differentially expressed transcription factors (TFs) that could control the expression of cell cycle-, cell division-, and cell proliferation-related DEGs mentioned above (all genes in **Figure 2A**). TF–target interactions in the ESCAPE, TRRUST, and ENCODE database were integrated, which provides more comprehensive information. Network hub analysis found that E2F1, EZH2, and EGR1 can target at least five genes (degree ≥5). The degree of the node E2F1 is the largest, suggesting that it could drive the expression of most downstream target genes in LUAD (**Figure 6A**). Pearson correlation coefficient analysis showed that *E2F1* was closely and positively correlated with *MYBL2*, *TYMS*, *FOXM1*, *CHAF1B*, *NDC80*, *PRC1*, *CDK1*, *AURKB*, *CCNB1*, *EZH2*, *DDX11*, *NUSAP1*, *CDKN2A*, and *FEN1* (**Figure 6B**). The expression of E2F1 is much higher in LUAD than in normal samples, as confirmed by GEPIA2 (**Figure 6C**). TF E2F1 reportedly could bind to the promoter region of a number of genes that are involved in cell cycle regulation (54). It can mediate both cell proliferation (55) and TP53/p53-dependent apoptosis (56). Besides, a previous study showed that E2F1 could be a common marker gene of cell proliferation in various cancers (8). We thus speculate that E2F1 should also play crucial roles in LUAD *via* controlling the expression of cell cycle-, cell division-, and cell proliferation-related DEGs. Moreover, its consistent higher expression pattern in cancer samples compared with normal samples was conserved in various cancers, as confirmed by the DiffExp module of the Tumor IMmune Estimation Resource (TIMER, https://cistrome.shinyapps.io/timer/) (57) (**Figure 7**). Therefore, we conclude that E2F1 could be a driving factor in human LUAD, and the molecular mechanism may be involved in the regulation of cell cycle-, cell division-, and cell proliferation-related DEGs.

### Bioactive Inhibitors for the Treatment of LUAD

**Figure 8:**
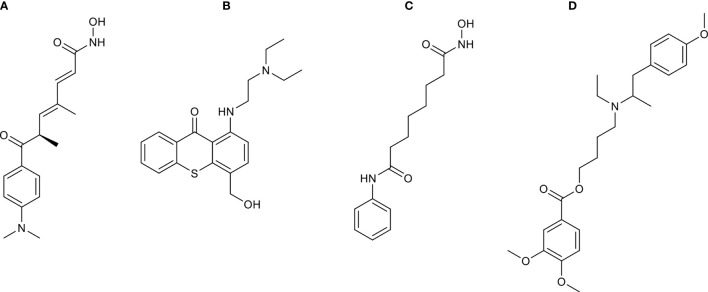
The 2D structures of compounds identified by cMAP analysis. **(A)** Trichostatin A, **(B)** hycanthone, **(C)** vorinostat, and **(D)** mebeverine.

To discover potential bioactive molecules/compounds that could suppress cell proliferation of LUAD, the probe set lists of upregulated genes (*CDC20*, *E2F1*, *TPX2*, *CENPF*, *BUB1*, *PLK1*, *KIF2C*, *AURKB*, *CDKN3*, *BUB1B*, *HMGA2*, *CDK1*, *ASPM*, and *CKS1B*) and downregulated (*TACC1* and *MYH10*) genes were loaded into the cMAP web server as query signatures. This resulted in four promising therapeutic agents for LUAD, including trichostatin A, hycanthone, vorinostat, and mebeverine (**Table 2**). The strong negative scores indicated a suppressing effect of all bioactive compounds, consistent with the negative connection with upregulated genes and positive connection with downregulated genes (**Table 2**). The 2D structures of these compounds were retrieved from PubChem (https://pubchem.ncbi.nlm.nih.gov), as shown in **Figure 8**. All four promising therapeutic agents have not been approved in LUAD treatment.

**Table 2:** Four bioactive compounds identified as potential therapeutic agents for LUAD.

CMAP name	Dose	Score	Up	Down
Trichostatin A	100 nM	−1.000	−0.475	0.787
Hycanthone	11 µM	−0.996	−0.552	0.705
Trichostatin A	1 µM	−0.965	−0.466	0.752
Vorinostat	10 µM	−0.929	−0.315	0.858
Mebeverine	9 µM	−0.914	−0.366	0.787

Note that only the results with scores less than 0.900 were shown.

## Discussion

Cell proliferation is one of the major reasons responsible for tumor recurrence, metastasis, and resistance to cancer therapy (7). Targeting cell proliferation special genes should be an ideal strategy for cancer therapy. Various targets were found for cancer therapy methods, such as chimeric antigen receptor T (CAR-T), cytokine-induced killer (CIKs), immune checkpoint blockade (anti-PD), and tumor-infiltrating lymphocytes (TILs). For example, EGFR and CD19 were considered ideal antigens for CAR-T cell therapy (58), and PD-1, PD-L1, and PD-L2 were usually adopted in anti-PD therapy (3). However, as ever more therapeutic targets are emerging and given the imperfect effect of these therapeutic methods, there is increasingly more attention to be paid to the cell proliferation-associated genes. Therefore, a systemic study of the molecular mechanism of the cell cycle-, cell division-, and cell proliferation-associated genes in LUAD and identification of relevant diagnostic and prognostic biomarkers would expand our knowledge for the treatment of LUAD and facilitate clinical diagnosis.

Based on both tissues and cells, we identified 434 common upregulated and 828 common downregulated DEGs between cancer and normal samples. The common upregulated DEGs were significantly associated with the cell cycle-, cell division-, and metabolism-related biological processes and pathways in LUAD. This close correlation suggesting the cell proliferation process is some material and energy dependent. A block of the material and energy metabolic pathways should be a novel strategy to slow down LUAD development. On the contrary, genes in cell adhesion, immune response, and inflammatory response-related biological processes and pathways were suppressed. An improvement of the activity of these genes should greatly reduce the mobility of cancer cells, slow down cancer cell transfer, and increase the resistibility of the human body. The gene signature between oncogene-addicted (EGFR/ALK/ROS1 mutation) LUAD and wild-type LUAD may be slightly different. However, due to the insufficient mutation information, this work is laid aside for the moment.

Notably, 11 common upregulated DEGs (*TPX2*, *CENPF*, *BUB1*, *PLK1*, *KIF2C*, *AURKB*, *CDKN3*, *BUB1B*, *CDK1*, *ASPM*, and *CKS1B*) involved in all three biological processes (cell cycle, cell division, and cell proliferation) could be used as prognostic markers for LUAD, namely, 9 previously reported prognostic markers (*TPX2*, *CENPF*, *BUB1*, *PLK1*, *KIF2C*, *AURKB*, *BUB1B*, *CDK1*, and *ASPM*) and 2 promising prognostic markers (*CDKN3* and *CKS1B*). These genes play crucial roles in the pathogenesis and development of various cancers. For example, *TPX2* promotes metastasis in LUAD (59). *BUB1*, *BUB1B*, *PLK1*, and *CDC20* were suggested as LUAD stem cell biomarkers (60). *CENPF*, *KIF2C*, *CDK1*, *ASPM*, and *MYH10* were also proposed as diagnosis and prognosis biomarkers of LUAD (61–66). *CDKN3* was not yet reported as a diagnosis or prognosis biomarker but expected to be a promising therapeutic target in LUAD (63). *CKS1B* was also reported as a potential target to improve cancer therapy (67). OS analysis showed that a higher expression of the 11 common upregulated genes always associated with a significantly shorter life cycle. In other words, the higher expressions of these genes are risk factors for LUAD patients, which could be used as indicators for diagnosis and prognosis. Also, *PLK1* could be a promising therapeutic target for LUAD treatment. All these demonstrate that these cell cycle-, cell division-, and cell proliferation-associated genes are of vital importance to the pathogenesis and development of LUAD, and targeting these genes should be new cues for cancer therapy.

Network analysis identified key regulators associated with cell cycle and cell division in LUAD. Network hub analysis revealed that 12 genes have a large number of connections with other genes, namely, *CDK1*, *CCNB1*, *PLK1*, *CCNA2*, *CDC20*, *CDKN2A*, *AURKA*, *CDC5L*, *AURKB*, *SNW1*, *MCM2*, and *CDK2*. Network module analysis showed most CDC (*CDC6*, *CDC20*, *CDC45*, *CDCA5*, *CDCA7*, and *CDCA8*)- and CDK (*CDK1*, *CDKN1C*, *CDKN2A*, and *CDKN3*)-associated genes were obviously enriched and closely interacted with each other. Most CDC- and CDK-associated genes were increased in LUAD, including the hub genes *CDK1* and *CDC20*, suggesting the important roles of these genes. Some CDK inhibitors have been approved by the FDA and used in cancer treatment. For example, ribociclib targeting CDK4/6 is used in breast cancer therapy (68). However, whether CDK1 or other CDK-associated genes can be targets of the inhibitors (such as abemaciclib, palbociclib, and ribociclib) used in LUAD treatment is currently not clear. Furthermore, we found that these cell cycle- and cell division-associated genes may cooperate with some transcription regulators, such as *TP53* and *MYC* with known roles in cancer, to promote the cell proliferation and progress of LUAD. More future work will be done to understand the detailed molecular mechanisms.

We also found that E2F1 could be a potential regulator that controls the expression of many cell cycle-, cell division-, and cell proliferation-related DEGs (**Figure 6A**). The increased expression of E2F1 is conserved in almost all cancers (**Figure 7**), indicating a similar role of E2F1 in these cancers from an evolutionary point of view. These findings should be of particular importance because most downstream genes are consistently and positively correlated with E2F1, so they can be mediated only by controlling the expression of E2F1. Hopefully, the discovery of chemicals such as small-molecule drugs and miRNAs that especially target E2F1 may be an effective treatment strategy for cancer therapy. Similarly, we supposed that some other genes (especially the TFs) with conserved expression patterns among various cancers may also have vital roles in LUAD, and more attention will be paid to these genes in the future.

Based on the key regulators involved in cell proliferation, we identified four therapeutic agents (trichostatin A, hycanthone, vorinostat, and mebeverine) for the treatment of LUAD. Trichostatin A is a histone deacetylase inhibitor, which was reported to inhibit breast cancer and glioma proliferation by inducing cell cycle arrest and apoptosis (69, 70). A similar effect was also reported for hycanthone, vorinostat, and mebeverine in other cancers (71–74). Combined utilization of these bioactive compounds could be a new therapeutic strategy that specifically inhibits the cell cycle-, cell division-, and cell proliferation-related genes in LUAD.

In summary, we detected DEGs between LUAD and normal lung samples using both tissues and cells. A total of 434 increased and 828 decreased genes were found in LUAD. Special attention was paid to cell cycle-, cell division-, and cell proliferation-associated genes. Eleven increased genes involved in cell cycle, cell division, and cell proliferation in LUAD can be used as prognostic markers. *PLK1* could be a promising therapeutic target for LUAD. Network analysis showed that *CDK1* and *CDC20* may play crucial roles in cell proliferation of LUAD. Also, we found that E2F1 can mediate the expression of most cell cycle-, cell division-, and cell proliferation-related DEGs. This work revealed key regulators that contribute to cell proliferation in LUAD and identified four potential therapeutic agents for treatment strategy.

## Data Availability Statement

The original contributions presented in the study are included in the article/**Supplementary Material**. Further inquiries can be directed to the corresponding authors.

## Ethics Statement

The studies involving human participants were reviewed and approved by the Ethics Committee of Shanghai Chest Hospital [KS1740]. Written informed consent for participation was not required for this study in accordance with the national legislation and the institutional requirements. Ethical review and approval was not required for the animal study.

## Author Contributions

KW, RL, CP, and LL designed the study and revised the manuscript. KW, MZ, and JW wrote, analyzed, and interpreted the data. PS carried out qRT-PCR experiment. PS, JL, and HJ revised the manuscript. All authors contributed to the article and approved the submitted version.

## Funding

This work was supported by a special project in the framework of the National Key Research and Development Program (NKRDP) (2017YFA0104500, 2017YFA0104600, 2020YFA010045), National Natural Science Foundation of China (31370904, 31601163, 81671579, 81671579, 81773273, 82071856), National and Provincial Multi-disciplinary Cooperative Diagnosis and Treatment Capacity Building Project for Major Diseases (Lung Cancer), Shanghai Municipal Science and Technology Major Project (2018SHZDZX01), Shanghai Science and Technology Commission Experimental Animal Research Project (18140903800), Shanghai Pujiang Program (15PJD021), Program for scientific and technological innovation from the Science and Technology Commission of Shanghai Municipality (15401900500, 18140903800), Shuguang Planning of Shanghai Municipal Education Commission of (16SG14), Program of Shanghai translational medicine collaborative innovation center cooperation (TM201522, TM201721), Shanghai Municipal Commission of Health, Scientific Research Program of Traditional Chinese medicine (2020JP009), Shanghai Jiao Tong University Affiliated Chest Hospital Major Project (YJXT20190106), and Shanghai Transportation Collaborative Self-help Project of Collaborative Innovation Center of University Translational Medicine (TM201522, TM201721).

## Conflict of Interest

The authors declare that the research was conducted in the absence of any commercial or financial relationships that could be construed as a potential conflict of interest.

## Publisher’s Note

All claims expressed in this article are solely those of the authors and do not necessarily represent those of their affiliated organizations, or those of the publisher, the editors and the reviewers. Any product that may be evaluated in this article, or claim that may be made by its manufacturer, is not guaranteed or endorsed by the publisher.

## Supplementary Material

Supplementary Figure 1 The heatmap of DEGs involved in the cell cycle. All DEGs with the absolute FC of ≥ 2.0 and p-value of ≤ 0.05 were showed.

Supplementary Figure 2 The heatmap of DEGs involved in cell division. All DEGs with the absolute FC of ≥ 2.0 and p-value of ≤ 0.05 were showed.

Supplementary Figure 3 The heatmap of DEGs involved in cell proliferation. All DEGs with the absolute FC of ≥ 2.0 and p-value of ≤ 0.05 were showed.

Supplementary Table 1 List of the common DEGs in both patient tissues and cell lines between human lung cancer cells and adjacent lung normal cells.

Supplementary Table 2 Enriched functions of the common DEGs *via* DAVID Gene Ontology annotation.
