# Supplementary figures and images for: A Systematic Analysis Identifies Key Regulators Involved in Cell Proliferation and Potential Drugs for the Treatment of Human Lung Adenocarcinoma
Source: Front Oncol. 2021 Sep 28;11:737152. doi: 10.3389/fonc.2021.737152 (PMC8505978; doi:10.3389/fonc.2021.737152)

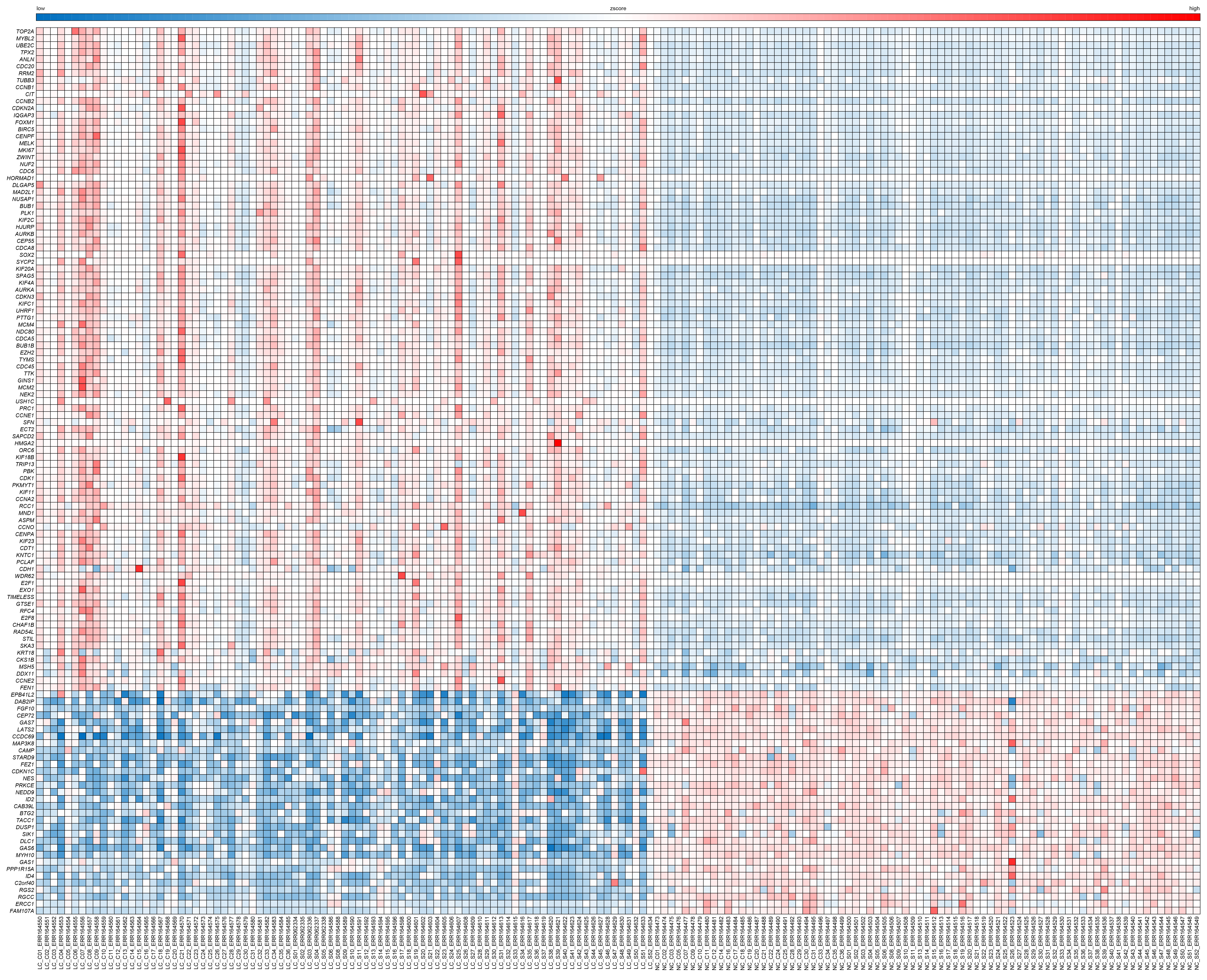

Supplement: Supplementary Figure 1 — The heatmap of DEGs involved in the cell cycle. All DEGs with the absolute FC of ≥ 2.0 and p-value of ≤ 0.05 were showed. [file Image_1.tif]

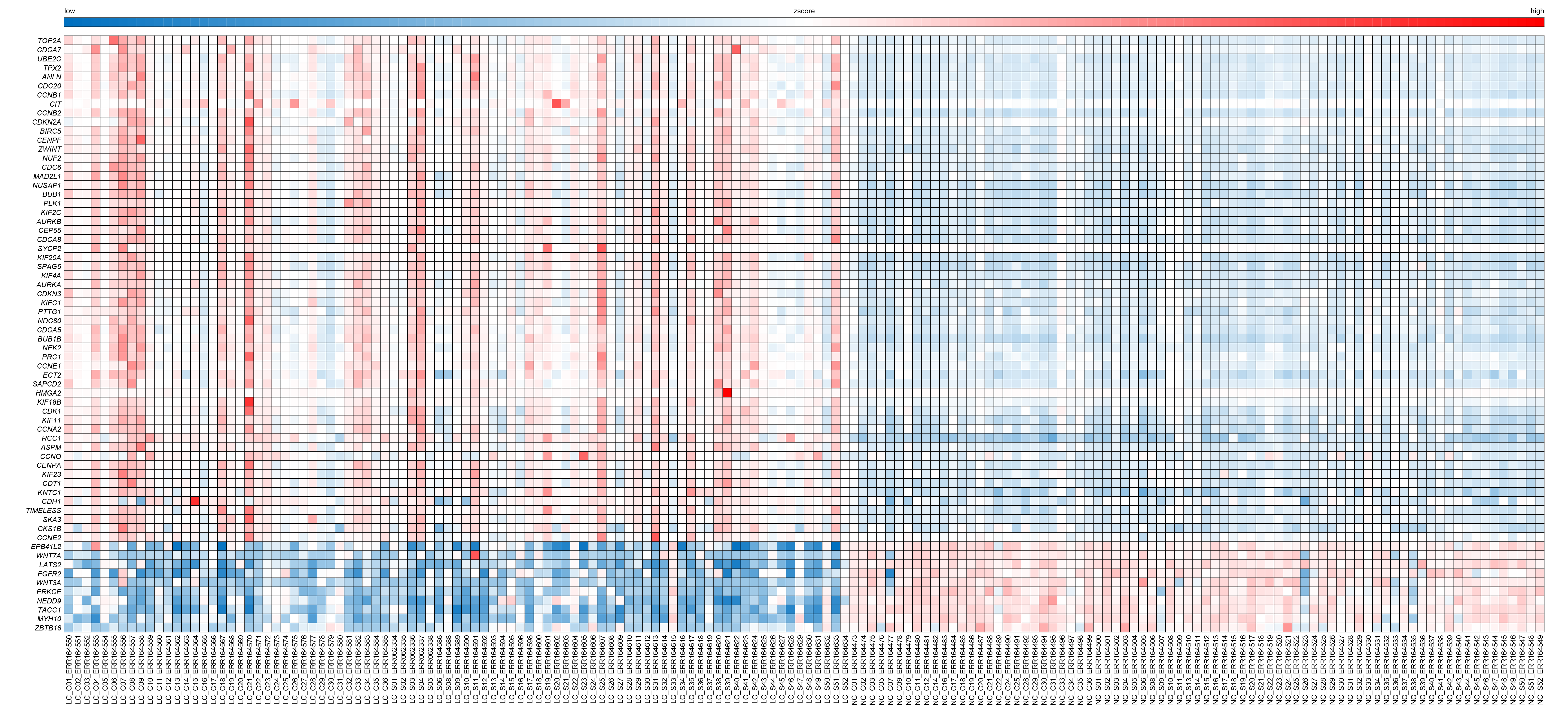

Supplement: Supplementary Figure 2 — The heatmap of DEGs involved in cell division. All DEGs with the absolute FC of ≥ 2.0 and p-value of ≤ 0.05 were showed. [file Image_2.tif]

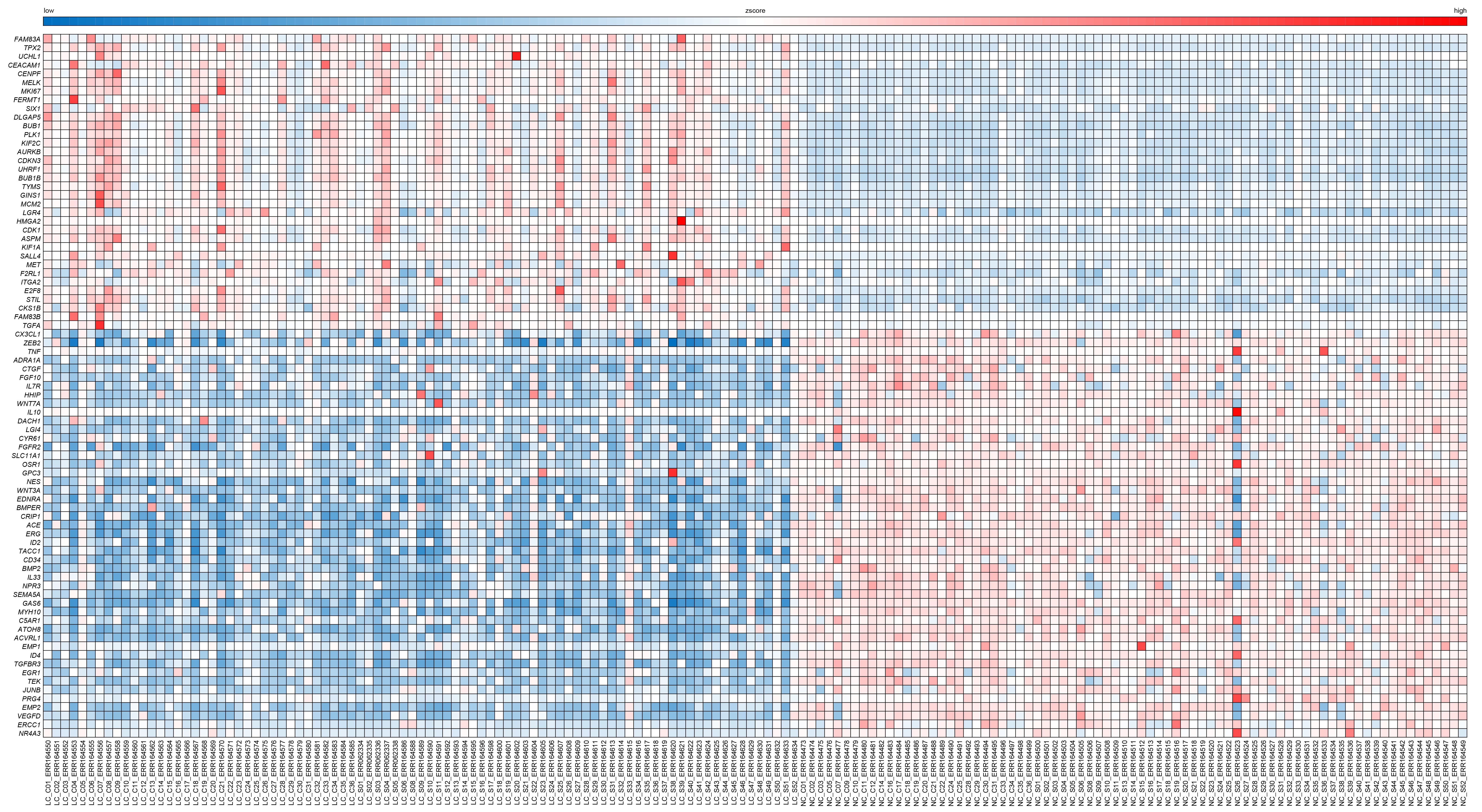

Supplement: Supplementary Figure 3 — The heatmap of DEGs involved in cell proliferation. All DEGs with the absolute FC of ≥ 2.0 and p-value of ≤ 0.05 were showed. [file Image_3.tif]
